# Assessment of the causal effect of lifestyle factors on postoperative infections based on Mendelian randomization

**DOI:** 10.1097/MD.0000000000046281

**Published:** 2025-12-12

**Authors:** Wen-Juan Fu, Xin-Xin Ni, Hong Yang, Wei Lian, Feng Xu

**Affiliations:** a Hospital Infection Management Section, Wujin Affiliated Hospital of Nanjing University of Traditional Chinese Medicine, Changzhou, P.R. China; b Wuxi Huishan People’s Hospital, Wuxi, P.R. China; c Wuxi Maternal and Child Health Hospital, Wuxi School of Medicine, Jiangnan University, Wuxi, P.R. China.

**Keywords:** Mendelian randomization, modifiable lifestyle factors, postoperative infection, risk factors, single nucleotide polymorphisms

## Abstract

Determining causal relationships between modifiable lifestyle factors and postoperative infections is crucial for guiding preventive strategies, yet establishing causality in observational studies remains challenging. This study aimed to investigate whether educational attainment, sleep duration, alcohol consumption, smoking, and body mass index (BMI) are causally linked to postoperative infection risk using Mendelian randomization (MR). We conducted a 2-sample MR analysis utilizing genome-wide association study data for 5 modifiable lifestyle factors as exposures and 2 postoperative infection genome-wide association study datasets as outcomes. Primary causal estimates were derived using inverse-variance weighted (IVW), with sensitivity analyses performed via MR-Egger regression, weighted median, and Mendelian randomization pleiotropy residual sum and outlier methods to assess pleiotropy and outliers. Heterogeneity was evaluated using Cochran *Q* statistic, and reverse MR analyses were employed to test for reverse causation. All analyses were implemented in R with rigorous statistical thresholds. BMI exhibited a positive causal association with postoperative infection risk [IVW method: dominance ratio (odds ratio) = 1.002, 95% CI (1.001–1.003), *P* = 2.60E‐05], while higher educational attainment (years of schooling) was inversely associated [IVW method: odds ratio = 0.998, 95% CI (0.997–1.000), *P* = .009]. Sensitivity analyses confirmed robustness, with no evidence of horizontal pleiotropy or influential outliers. No significant associations were observed for sleep duration, alcohol use, or smoking. This first genome-wide MR study demonstrates that elevated BMI increases, and longer education decreases, the risk of postoperative infections. These findings underscore BMI and education as modifiable targets for infection prevention, providing a foundation for clinical and public health interventions.

## 1. Introduction

Postsurgical infections are surgical site or systemic infections that occur after a surgical operation due to a variety of reasons such as surgical trauma, implantation of foreign objects, and decreased immunity. Such infections may involve the skin, soft tissues, organs, or blood, and may be life-threatening in severe cases.^[1,2]^ Infections have been reported to occur in 8.5% of initial surgeries and 12% of revision surgeries.^[3]^ Previous retrospective studies have identified several risk factors for the development of postoperative infections, such as diabetes mellitus, advanced age, obesity, poor nutritional status, multilevel surgery, prolonged operative time, and high blood loss.^[4]^ Therefore, exploring the risk factors for postoperative infections will provide a basis for developing postoperative infection prevention measures.

Mendelian randomization (MR) is a powerful approach for assessing causal relationships between exposures and outcomes. It exploits the random distribution of genetic variation during gametogenesis to reduce chance bias and avoid confounding and misleading associations observed in observational studies.^[5]^ Compared to randomized controlled trials, MR studies often require fewer resources and shorter follow-up periods.^[6]^ Given that large-scale randomized controlled trials can be resource-intensive and bound by strict experimental conditions, MR methods have emerged as a valuable tool for probing biological mechanisms.

Numerous studies have established a clear link between obesity, blood lipids and postoperative infections.^[7]^ The causal relationship between increased body composition, encompassing body mass index (BMI) and waist circumference, and the occurrence of surgical incision infections has been demonstrated, underscoring the importance of preventing surgical incision infections in obese patients.^[8]^ Clinical and Mendelian randomization studies indicate that obesity, as measured by BMI, is associated with the development of postoperative incisional hernia and infection.^[9]^ Further research is still warranted to mitigate the incidence of surgical incision infections in obese patients. However, current research remains unable to determine whether any modifiable lifestyle factors beyond BMI are associated with postoperative infection. Therefore, we evaluated whether modifiable lifestyle factors—including educational attainment, smoking, body mass index, sleep duration, and alcohol consumption—constitute causal risk factors for postoperative infection.

## 2. Materials and methods

### 2.1. Study design

**Figure 1.**
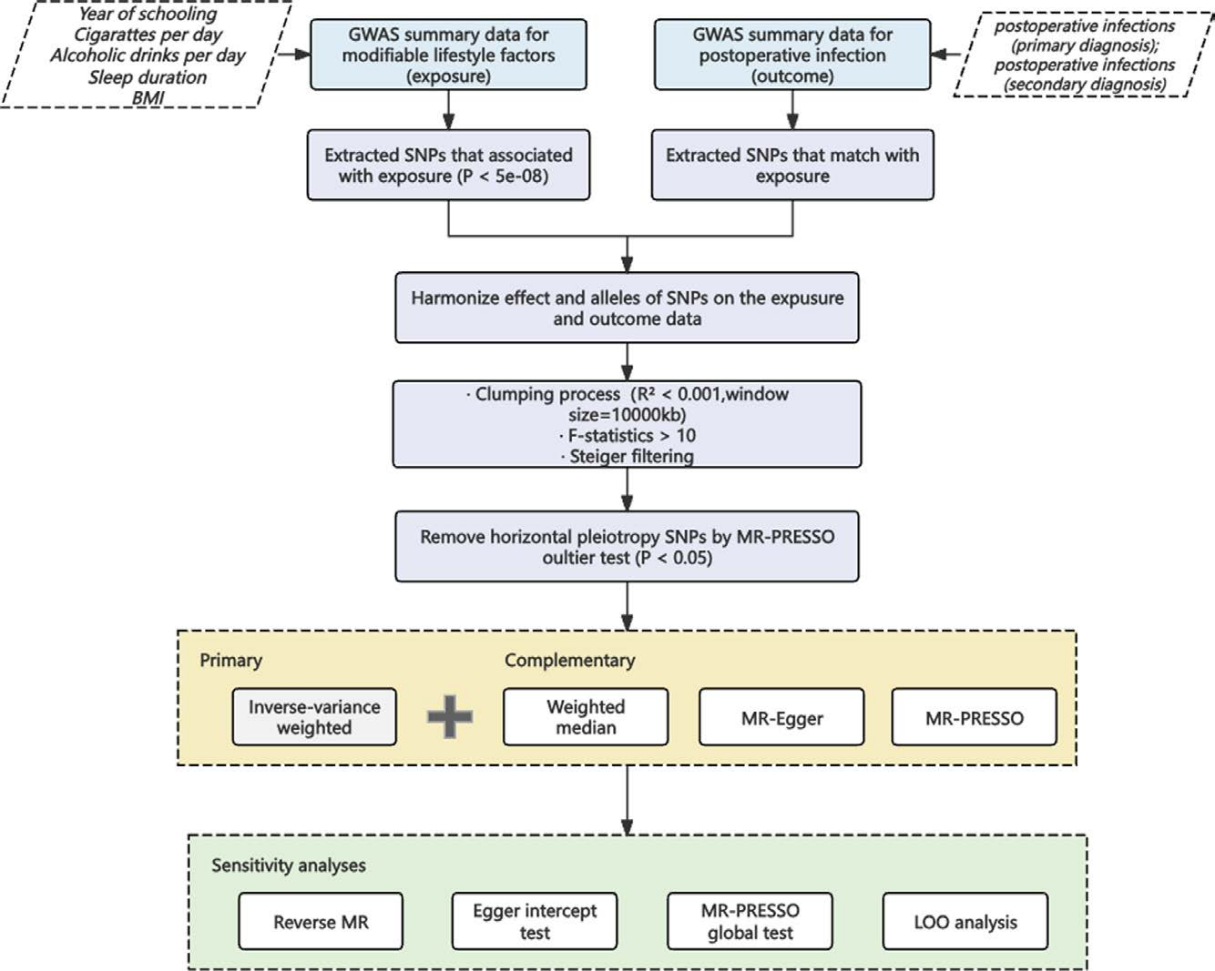
Overall study design and workflow. GWAS = genome-wide association study, LOO = leave-one-out, MR-PRESSO = Mendelian randomization pleiotropy residual sum and outlier, SNPs = single nucleotide polymorphisms.

In this study, we considered each modifiable lifestyle factor as an exposure factor and the risk of postoperative infection as an outcome. As shown in Figure 1, the entire study workflow was as follows: we used single nucleotide polymorphisms (SNPs) significantly associated with exposure as instrumental variables (IVs). To ensure the robustness of MR studies, each IV should satisfy the following conditions: significantly associated with exposure, independent of all other IVs and potential confounders, and affecting the outcome only by influencing exposure.^[10,11]^ We ensured the validity of the IV by estimating the *F*-statistic and performing the Steiger test; Egger regression and Mendelian randomization pleiotropy residual sum and outlier (MR-PRESSO) methods were used to detect and calibrate horizontal pleiotropy and outliers; leave-one-out (LOO) analyses were performed to assess the presence of the primary IV; and reverse MR analyses were performed to determine the likelihood of reverse causality.^[12–14]^

### 2.2. Genome-wide association study (GWAS) data for exposure and outcome

We performed a 2-sample MR analysis using summary statistics from the largest publicly available genome-wide association studies for educational attainment (year of schooling), sleep duration, smoking (cigarettes per day), alcohol consumption (alcoholic drinks per week) and BMI. GWAS summary data on postoperative infections (primary diagnosis) and postoperative infections (secondary diagnosis) were obtained from results of the GWAS on the UK Biobank. We evaluated for postoperative infection using International Classification of Diseases, Tenth Revision, Clinical Modification (ICD-10-CM) codes. These outcomes were chosen to not only capture immediate postoperative outcomes, but also potential long-term consequences of surgical interventions, including those not present on the initial admission.^[9,15]^ The characteristics of the summary datasets were shown in Table S1, Supplemental Digital Content, https://links.lww.com/MD/Q792.

### 2.3. Selection of instrumental variables

We first screened nonallelic SNPs from the 1000 Genomes Project for minor allele frequency > 0.05 and significantly associated with modifiable lifestyle factors (*P* < 5E‐08 in GWAS) and performed a 1000 kb window for linkage disequilibrium (LD) cluster analysis to ensure that the selected SNPs were independent of each other (pairwise LD *r*^2^ < 0.001). Subsequently, the variance explained by each SNP was calculated (*r*^2^ = (2β^2^ × EAF × (1 − EAF))/(2β^2^ × EAF × (1 − EAF) + 2N × EAF × (1 − EAF) × SE^2^)) and the *F*-statistic (*F* = (*R*^2^ × (N − 2))/(1 − *R*^2^)), where EAF is the effect allele frequency, β and SE represent the effect size and the standard error, and N is the GWAS sample size.^[16]^ Only modifiable lifestyle factors with at least 3 valid IVs were retained for subsequent MR analysis after excluding weak IVs with *F*-statistics < 10 and abnormal SNPs identified by the MR-PRESSO outlier test (*P* < .05).

### 2.4. MR analysis

We used the inverse-variance weighted (IVW) method, which estimates causal effects by integrating the Wald ratios of all genetic variants, as the main analytical method, with the underlying assumption that there is no horizontal pleiotropy in all IVs.^[17]^ To check the robustness of the results, 3 complementary analytical methods were also used: the weighted median method to reduce the potential bias introduced by some of the invalid instrumental variables by assigning higher weights to the valid IVs^[18]^; the MR-Egger regression to detect and correct for the effect of possible horizontal pleiotropy by introducing an intercept term; and the MR-PRESSO method to identify and remove outliers, thus optimizing the final causal effect estimates.^[14]^ Through this multi-method strategy, we are able to comprehensively assess the impact of potential pleiotropy and outliers on the analytical results, significantly improving the reliability of causal inference.

### 2.5. Complementary, sensitivity, and reverse MR analyses

In order to check the robustness of the significant associations found by the IVW method, we conducted a comprehensive supplementary analysis and sensitivity tests. Instrumental variable heterogeneity was first assessed using Cochran *Q* test, while potential horizontal pleiotropy was detected using the MR-Egger intercept test and the MR-PRESSO global test. In addition, we performed LOO analyses to identify dominant IVs that might have an excessive impact on the results.^[19]^ Given the sample size advantage of the cancer GWAS, we adopted more stringent IV screening criteria with reference to previous studies^[20]^: selecting IVs that were independent of each other (paired LD *r*² < 0.001) within a window of 10,000 kb and that met the genome-wide threshold for significance (*P* < 5E‐05) SNPs as instrumental variables. Finally, we recognized associations estimated by the IVW method with *P* < .05 as statistically significant. This rigorous series of analytical procedures ensured the reliability and robustness of the results.

## 3. Results

### 3.1. Genetic IVs

A total of 899 associations between 2,360,632 common genetic variants (minor allele frequency > 0.05) at the LD clumping criteria of pairwise LD *r*^2^ < 0.001 within a 10,000 kb window and the significance threshold of *P* < 5E‐08 were obtained.^[21]^ After a rigorous screening process to exclude weak IVs and outliers, which included criteria such as *F*-statistics < 10 and Steiger test, in addition to the application of the MR-PRESSO outlier test with a significance level of *P* < .05, the subsequent MR analyses were conducted with a refined set of metabolites. The detailed information on the IVs selected for downstream MR analyses is shown in Table S1, Supplemental Digital Content, https://links.lww.com/MD/Q792.

### 3.2. Effects of modifiable lifestyle factors on postoperative infection

The use of these IVs enabled the estimation of the causal association between the 5 modifiable lifestyle factors and 2 postoperative infections. This analysis identified a total of 4 suggestive associations (*P* < .05). The MR analysis demonstrated that year of schooling was inversely associated with postoperative infection (primary diagnosis) [IVW method: odds ratio [OR] = 0.996, 95%CI(0.995–0.998), *P* = 2.18E‐07; BMI was positively associated with postoperative infection (primary diagnosis) [IVW method: OR = 1.003, 95% CI (1.002–1.004), *P* = 1.65E‐10]; sleep duration was inversely associated with postoperative infection (primary diagnosis) [IVW method: OR = 0.994, 95% CI (0.994–0.998), *P* = .0015]. Genetically predicted schooling was inversely associated with postoperative infection (secondary diagnosis) [IVW method: OR = 0.998, 95%CI (0.997–1.000), *P* = .009; BMI was positively associated with postoperative infection (secondary diagnosis) [IVW method: OR = 1.002, 95% CI (1.001–1.003), *P* = 2.60E‐05]; sleep duration was inversely associated with postoperative infection (secondary diagnosis) [IVW method: OR = 0.996, 95% CI (0.992–1.000), *P* = .04] (shown in Table 1).

**Table 1:** Significant associations identified in primary MR analyses using the inverse-variance weighted (IVW) method.

Phenotype	Number of IVs		IVW		MR-Egger		Weighted median		Weighted mode		MR-PRESSO	
			OR (95% CI)	*P*	OR (95% CI)	*P*	OR (95% CI)	*P*	OR (95% CI)	*P*	OR (95% CI)	*P*
Diagnoses—main ICD10: T81.4 Infection following a procedure, not elsewhere classified												
Year of schooling		261	0.996 (0.995–0.998)	**2.18E‐07**	1.003 (0.997–1.009)	.347	0.997 (0.995–0.999)	**.011**	0.998 (0.991–1.004)	.450	0.996 (0.995–0.998)	**1.05E‐06**
Cigarettes per day		17	1.001 (0.999–1.002)	.370	0.999 (0.997–1.001)	.473	1.000 (0.998–1.001)	.747	1.000 (0.998–1.001)	.730	1.001 (0.999–1.002)	**3.64E‐01**
Alcoholic drinks per week		27	1.000 (0.996–1.004)	.979	1.001 (0.985–1.017)	.927	1.001 (0.995–1.007)	.825	1.002 (0.993–1.011)	.692	1.000 (0.996–1.004)	**9.80E‐01**
BMI		360	1.003 (1.002–1.004)	**1.65E‐10**	1.002 (0.999–1.005)	.192	1.004 (1.002–1.005)	**9.93E‐06**	1.003 (1.000–1.006)	**.023**	1.003 (1.002–1.004)	**1.27E‐09**
Sleep duration		60	0.994 (0.990–0.998)	**.001**	1.005 (0.988–1.022)	.596	0.992 (0.987–0.998)	**.004**	0.986 (0.974–0.998)	**.024**	0.994 (0.991–0.998)	**.003**
Diagnoses—secondary ICD10: T81.4 Infection following a procedure, not elsewhere classified												
Year of schooling		194	0.998 (0.997–1.000)	**.009**	1.000 (0.994–1.006)	.987	0.998 (0.996–1.001)	.149	1.000 (0.995–1.005)	.882	0.998 (0.997–1.000)	**.013**
Cigarettes per day		14	1.000 (0.999–1.001)	.655	1.000 (0.999–1.002)	.559	1.000 (0.999–1.001)	.669	1.000 (0.999–1.001)	.636	1.000 (0.999–1.001)	.599
Alcoholic drinks per week		22	1.000 (0.997–1.004)	.989	1.005 (0.993–1.018)	.426	0.999 (0.994–1.004)	.699	1.000 (0.993–1.007)	.934	1.000 (0.997–1.003)	.988
BMI		289	1.002 (1.001–1.003)	**2.60E‐05**	1.001 (0.999–1.004)	.319	1.002 (1.000–1.003)	**.035**	1.002 (0.999–1.004)	.153	1.002 (1.001–1.003)	**1.75E‐05**
Sleep duration		51	0.996 (0.992–1.000)	**.040**	0.979 (0.950–1.009)	.183	0.996 (0.991–1.000)	.078	0.995 (0.985–1.005)	.338	0.996 (0.993–1.000)	**.053**

Bold values indicate *P* < .05.

BMI = body mass index, CI = confidence interval, IV = instrumental variable, IVW = inverse-variance weighted, MR = Mendelian randomization, OR = odds ratio.

### 3.3. Sensitivity analysis

**Figure 2.**
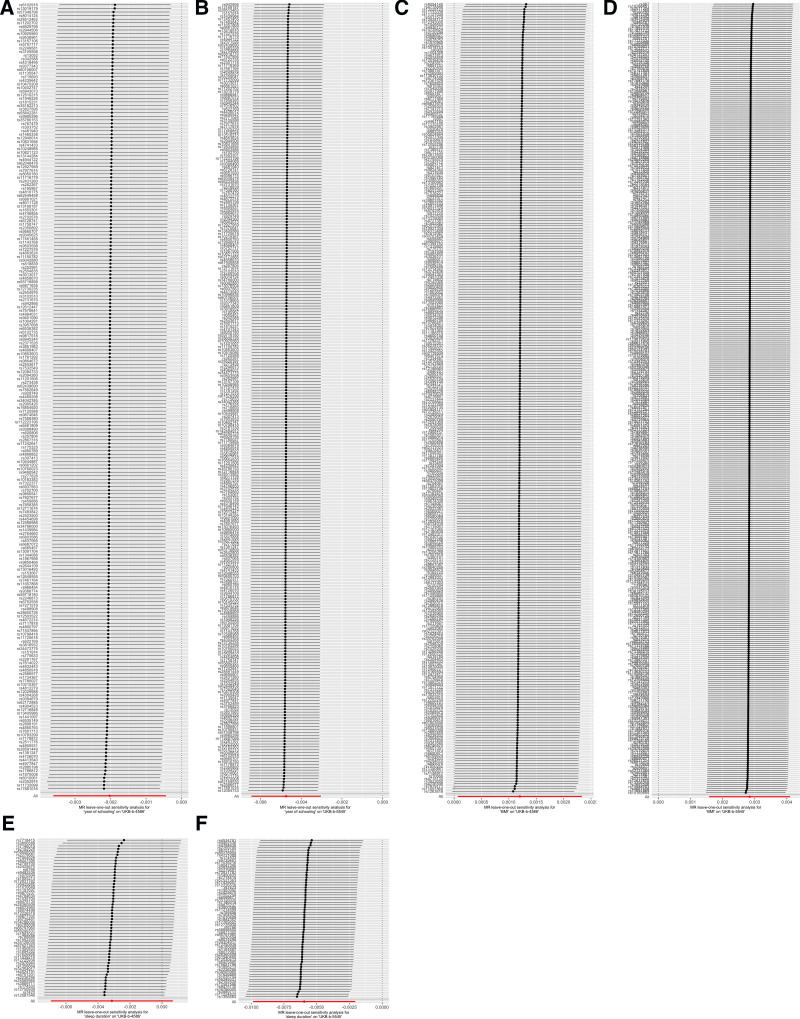
Scatter plot showing the genetic associations of modifiable lifestyle factors on risk of postoperative infections. (A) Year of schooling on postoperative infection (primary diagnosis); (B) Year of schooling on postoperative infection (secondary diagnosis); (C) BMI on postoperative infection (primary diagnosis); (D) BMI on postoperative infection (secondary diagnosis); (E) Sleep duration on postoperative infection (primary diagnosis); and (F) Sleep duration on postoperative infection (secondary diagnosis). BMI = body mass index, IV = instrumental variables.

**Figure 3.**
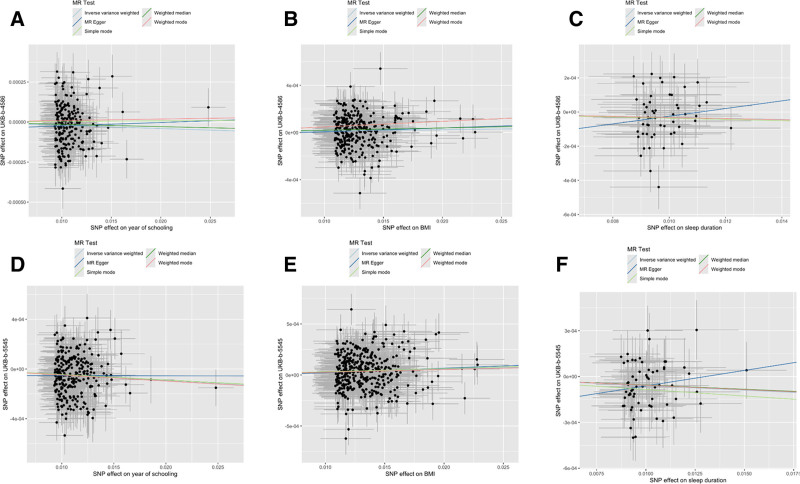
The funnel plot represents IVs for modifiable lifestyle factors on risk of postoperative infections. (A) Year of schooling on postoperative infection (primary diagnosis); (B) Year of schooling on postoperative infection (secondary diagnosis); (C) BMI on postoperative infection (primary diagnosis); (D) BMI on postoperative infection (secondary diagnosis); (E) Sleep duration on postoperative infection (primary diagnosis); and (F) Sleep duration on postoperative infection (secondary diagnosis). BMI = body mass index, IV = instrumental variables.

In general, causal associations were robust when statistical significance (*P* < .05) was observed in the other 4 MR tests (usually weighted median test, weighted mode test, MR-Egger test, and MR-PRESSO test). Consequently, sleep duration on postoperative infection (secondary diagnosis) was excluded. Sensitivity analyses were performed to avoid the horizontal pleiotropy for MR estimate. Table S2, Supplemental Digital Content, https://links.lww.com/MD/Q792 depicts the results of the sensitivity analyses for 3 modifiable lifestyle factors and 2 postoperative infections with significant causal associations. Similarly, the association between sleep duration and postoperative infection (primary diagnosis) exhibited a different pattern of association compared to the other MR methods. The MR-Egger intercept test and MR-PRESSO global test indicated that only 1 association was affected by horizontal multidirectionality: year of schooling on postoperative infection (primary diagnosis). The LOO analysis confirmed that no single IV was found to dominate any of the 6 associations in Figure 2. As illustrated in Figure 3, 2 significant associations identified using the IVW method exhibited disparate patterns of association among the results of the other 4 MR methods. For instance, the association between year of schooling, sleep duration and postoperative infection (primary diagnosis) exhibited a different pattern of association compared to the other MR methods. Additionally, no significant heterogeneity among the IVs was found in any of the 6 associations (*P* > .05 for heterogeneity). In the reverse MR analysis, 38 SNPs of postoperative infection (primary diagnosis) and 27 SNPs of postoperative infection (secondary diagnosis) were selected as IVs for year of schooling, BMI and sleep duration, respectively. BMI was excluded from the analysis of postoperative infection (primary diagnosis) due to a significant association (*P* = .0016). Table S3, Supplemental Digital Content, https://links.lww.com/MD/Q792 lists all results of sensitivity and ambiguity analyses in Mendelian randomization, and Table S4, Supplemental Digital Content, https://links.lww.com/MD/Q792 lists all results of sensitivity and ambiguity analyses in reverse Mendelian randomization.

## 4. Discussion

Postoperative infections are one of the most common categories of hospital-acquired infections and one of the most important and serious postoperative complications. In this 2-sample Mendelian randomization analysis, we analyzed the relationship between 5 modifiable life factors and the incidence of postoperative infections using data from the UK Biobank and IEU OpenGWAS database, and the summary of the results showed that BMI was positively correlated with the incidence of postoperative infections, whereas education level was were negatively correlated. The remaining factors including smoking, alcohol consumption and sleep duration were not considered to be associated with postoperative infections.

Postoperative infections include surgical site infections (SSIs) and other site infections occurring in the perioperative period, including gastrointestinal infections and respiratory infections.^[22]^ Risk factors that are usually considered to be responsible for postoperative infections include intraoperative asepsis, postoperative surgical care, long duration of surgery, advanced age, diabetes mellitus, high BMI, and inappropriate use of antibiotics in the perioperative period.^[23–25]^ However, there are still many problems that need to be solved. John et al refuted that SSIs are due to intraoperative contamination by studying the results of microbial cultures performed on surgical incisions and suggested that pathogens far away from the area of SSIs at the surgical site, such as those from the teeth, gingiva, and gastrointestinal tract, are ingested by the immune cells and spread through the bloodstream to the wound site to cause infections.^[26]^ On the other hand, with the development of macro-genomics and RNA sequencing technologies, we can more easily sequence microorganisms and analyze the pathways of microorganisms from the built environment and the hair and skin of healthcare workers to the patient’s incisions, making it easier to identify blocking points and design new therapies.^[27,28]^ However, we also note that current research has focused less on the patient’s own factors as well as modifiable life factors.

Body mass index is an important indicator of an individual’s nutritional status, and the World Health Organization defines abnormal BMI as below 18.5 and above 30. Abnormal BMI has been associated with problems in various systems, including the cardiovascular, respiratory, endocrine, gastrointestinal, skeletal-muscular, and renal systems.^[29]^ BMI Abnormal BMI causes problems based on 2 main aspects, and weakness caused by too low BMI and obesity caused by too high BMI. Obese patients may have problems with difficult surgical access and anesthesia-related complications.^[30,31]^ On the other hand, obesity is associated with poor surgical outcomes such as longer operative times, greater surgical bleeding, and higher rates of secondary surgery.^[32,33]^ Helen et al have shown that obesity is a major problem in patients with obesity through the analysis of META analysis of 64 obesity-related studies, found that overweight and obese class I, II, and III were more likely to have postoperative infections, VTE, and renal complications compared with normal BMI patients.^[34]^

In our present study, we found that BMI was positively correlated with the odds of postoperative infection. This may be the result of an impaired immune system response due to the interaction of immune cells with fat cells.^[35–37]^ Increased wound tension, retractor use and poor local microcirculation may also contribute to the increased risk of surgical wound infection in obese patients.^[38]^ However, it is worth noting that MR could not analyze the risk factors associated with obesity leading to surgical wound infections, and thus may need to be analyzed further in subsequent studies.

Educational attainment, as another important variable life factor, is also thought to be associated with a wide range of diseases, and higher educational attainment is currently protective against a range of diseases, including cardiovascular disease, respiratory disease, and type 2 diabetes mellitus.^[39,40]^ In the present study, we found that higher levels of education similarly reduced the incidence of postoperative infections, which is consistent with the conventional wisdom that this may be related to better protection of the wound and better understanding of the content of the caregiver’s teaching, but is unproven. Similarly, other factors may explain the association, including alcohol consumption, smoking, poverty, employment, diet, psychological factors, and access to health care,^[41–43]^ but are not further elaborated in this paper because the associated factors, other than smoking and alcohol consumption, are not represented in the GWAS database.

However, it is worth noting that despite the relevant findings of this study, several limitations remain. For instance, the results are primarily based on specific European cohorts and populations, necessitating caution when generalizing to other groups. Furthermore, while instrumental variable analysis was employed to assess causal relationships, this method carries specific assumptions and limitations, such as the selection of instruments and issues of multicollinearity. Consequently, future studies should expand sample sizes to validate the applicability and stability of these modifiable lifestyle factors across diverse populations and delve deeper into their underlying biological mechanisms. Overall, this study offers new perspectives and approaches for assessing and preventing postoperative infection risks, providing important references and insights for subsequent research. It is anticipated that future studies will focus more on these modifiable lifestyle factors, delving deeper into their mechanisms of action in postoperative infections to make greater contributions to their prevention and treatment.

## 5. Conclusions

In conclusion, in this paper, we analyzed the relationship between variable lifestyle and postoperative infections by establishing a 2-sample MR approach, and our conclusions showed that BMI was positively correlated with postoperative infection patterns, whereas education and postoperative infection patterns were negatively correlated, and that preventive measures targeting both risk factors, including improving social education and changing the way that has been to lower the patient’s body mass index, would be a control of postoperative Infections are reliably controlled.

Through IVW and multiple sensitivity analyses, it was conclusively determined that increased BMI increases the risk of postoperative infections (secondary diagnosis) and increased year of schooling decreases the risk of postoperative infections (secondary diagnosis). This is the first systematic Mendelian randomization analysis using genome-wide data to assess the causal relationship between modifiable lifestyle factors and postoperative infections, and provides preliminary evidence for the effect of BMI and educational level on postoperative infections.

## Acknowledgments

We thank all the consortium studies for making the summary association statistics data publicly available.

## Author contributions

**Conceptualization:** Hong Yang.

**Data curation:** Wen-Juan Fu, Xin-Xin Ni, Hong Yang, Wei Lian.

**Formal analysis:** Xin-Xin Ni, Wei Lian.

**Methodology:** Hong Yang, Wei Lian, Feng Xu.

**Project administration:** Wei Lian.

**Validation:** Hong Yang.

**Writing – original draft:** Wen-Juan Fu, Xin-Xin Ni, Hong Yang.

**Writing – review & editing:** Feng Xu.

## Supplementary Material

## References

[1] Goswami K Stevenson KL Parvizi J. Intraoperative and postoperative infection prevention. J Arthroplasty. 2020;35(3s):S2–8. 32046826 10.1016/j.arth.2019.10.061

[2] Tan T Lee H Huang MS. Prophylactic postoperative measures to minimize surgical site infections in spine surgery: systematic review and evidence summary. Spine J. 2020;20:435–47. 31557586 10.1016/j.spinee.2019.09.013

[3] Zhu H Liu X Wang Z. Infection rate in 1033 elective neurosurgical procedures at a university Hospital in South China. J Neurol Surg A Cent Eur Neurosurg. 2017;78:467–71. 28437812 10.1055/s-0037-1598658

[4] Konda SR Dedhia N Ganta AK Behery O Haglin JM Egol KA. Risk factors for gram-negative fracture-related infection. Orthopedics. 2022;45:91–6. 35021025 10.3928/01477447-20220105-04

[5] Richmond RC Davey Smith G. Mendelian randomization: concepts and scope. Cold Spring Harb Perspect Med. 2022;12:a040501. 34426474 10.1101/cshperspect.a040501 PMC8725623

[6] Smith GD Ebrahim S. ‘Mendelian randomization’: can genetic epidemiology contribute to understanding environmental determinants of disease? Int J Epidemiol. 2003;32:1–22. 12689998 10.1093/ije/dyg070

[7] Yang T Chen Z Cao D. Genetic correlations and causal associations between BMI, HDL-C, and postoperative infections: a two-sample Mendelian randomization study. Sci Rep. 2025;15:11834. 40195352 10.1038/s41598-025-95812-2 PMC11977256

[8] Yang J Zhang F Xue H. Genetically predicted body composition and risk of surgical site infection: a Mendelian randomization study. Surg Infect. 2025;26:95–103. 10.1089/sur.2024.133 39531261

[9] Robinson JR Carroll RJ Bastarache L. Association of genetic risk of obesity with postoperative complications using mendelian randomization. World J Surg. 2020;44:84–94. 31605180 10.1007/s00268-019-05202-9 PMC6925615

[10] Birney E. Mendelian randomization. Cold Spring Harb Perspect Med. 2022;12:a041302. 34872952 10.1101/cshperspect.a041302 PMC9121891

[11] Boef AG Dekkers OM le Cessie S. Mendelian randomization studies: a review of the approaches used and the quality of reporting. Int J Epidemiol. 2015;44:496–511. 25953784 10.1093/ije/dyv071

[12] Burgess S Thompson SG. Bias in causal estimates from Mendelian randomization studies with weak instruments. Stat Med. 2011;30:1312–23. 21432888 10.1002/sim.4197

[13] Verbanck M Chen C-Y Neale B Do R. Detection of widespread horizontal pleiotropy in causal relationships inferred from Mendelian randomization between complex traits and diseases. Nat Genet. 2018;50:693–8. 29686387 10.1038/s41588-018-0099-7 PMC6083837

[14] Bowden J Davey Smith G Burgess S. Mendelian randomization with invalid instruments: effect estimation and bias detection through Egger regression. Int J Epidemiol. 2015;44:512–25. 26050253 10.1093/ije/dyv080 PMC4469799

[15] Denny JC Ritchie MD Basford MA. PheWAS: demonstrating the feasibility of a phenome-wide scan to discover gene-disease associations. Bioinformatics. 2010;26:1205–10. 20335276 10.1093/bioinformatics/btq126 PMC2859132

[16] Cuezva JM Chen G Alonso AM. The bioenergetic signature of lung adenocarcinomas is a molecular marker of cancer diagnosis and prognosis. Carcinogenesis. 2004;25:1157–63. 14963017 10.1093/carcin/bgh113

[17] Pierce BL Burgess S. Efficient design for Mendelian randomization studies: subsample and 2-sample instrumental variable estimators. Am J Epidemiol. 2013;178:1177–84. 23863760 10.1093/aje/kwt084 PMC3783091

[18] Hartwig FP Davey Smith G Bowden J. Robust inference in summary data Mendelian randomization via the zero modal pleiotropy assumption. Int J Epidemiol. 2017;46:1985–98. 29040600 10.1093/ije/dyx102 PMC5837715

[19] Li Y Liu H Ye S. The effects of coagulation factors on the risk of endometriosis: a Mendelian randomization study. BMC Med. 2023;21:195. 37226166 10.1186/s12916-023-02881-z PMC10210381

[20] Long Y Tang L Zhou Y Zhao S Zhu H. Causal relationship between gut microbiota and cancers: a two-sample Mendelian randomisation study. BMC Med. 2023;21:66. 36810112 10.1186/s12916-023-02761-6 PMC9945666

[21] Surendran P Stewart ID Au Yeung VPW. Rare and common genetic determinants of metabolic individuality and their effects on human health. Nat Med. 2022;28:2321–32. 36357675 10.1038/s41591-022-02046-0 PMC9671801

[22] Bassetti M Eckmann C Giacobbe DR Sartelli M Montravers P. Post-operative abdominal infections: epidemiology, operational definitions, and outcomes. Intensive Care Med. 2020;46:163–72. 31701205 10.1007/s00134-019-05841-5

[23] Ilyas M Sharma S Gupta V. Immunoglobulin G4 disease-related retroperitoneal fibrosis: a series of five cases. SA J Radiol. 2024;28:2830. 38840828 10.4102/sajr.v28i1.2830 PMC11151408

[24] Xie J Li Y Qiu M Liu X Zhou S Jiang J. Risk factors and nursing countermeasures of postoperative pulmonary infection in patients with breast cancer: a retrospective analysis. Medicine (Baltim). 2021;100:e26952. 10.1097/MD.0000000000026952 PMC8448041 34664826

[25] Wang YY Hu S-F Ying H-M. Postoperative tight glycemic control significantly reduces postoperative infection rates in patients undergoing surgery: a meta-analysis. BMC Endocr Disord. 2018;18:42. 29929558 10.1186/s12902-018-0268-9 PMC6013895

[26] Alverdy JC Hyman N Gilbert J. Re-examining causes of surgical site infections following elective surgery in the era of asepsis. Lancet Infect Dis. 2020;20:e38–43. 10.1016/S1473-3099(19)30756-X PMC8019154 32006469

[27] Gilbert JA Lynch SV. Community ecology as a framework for human microbiome research. Nat Med. 2019;25:884–9. 31133693 10.1038/s41591-019-0464-9 PMC7410146

[28] Bennett-Guerrero E Pappas TN Koltun WA. Gentamicin-collagen sponge for infection prophylaxis in colorectal surgery. N Engl J Med. 2010;363:1038–49. 20825316 10.1056/NEJMoa1000837

[29] Parratte S Pesenti S Argenson JN. Obesity in orthopedics and trauma surgery. Orthop Traumatol Surg Res. 2014;100(1 Suppl):S91–7. 24461910 10.1016/j.otsr.2013.11.003

[30] Mari A Abufaraj M Mansy K Sievert K-D. Obesity and its implications on nononcological urological surgery. Curr Opin Urol. 2017;27:456–63. 28650868 10.1097/MOU.0000000000000430

[31] Jiang J Teng Y Fan Z Khan S Xia Y. Does obesity affect the surgical outcome and complication rates of spinal surgery? A meta-analysis. Clin Orthop Relat Res. 2014;472:968–75. 24146361 10.1007/s11999-013-3346-3 PMC3916601

[32] Alimi M Hofstetter CP Pyo SY Paulo D Härtl R. Minimally invasive laminectomy for lumbar spinal stenosis in patients with and without preoperative spondylolisthesis: clinical outcome and reoperation rates. J Neurosurg Spine. 2015;22:339–52. 25635635 10.3171/2014.11.SPINE13597

[33] Myung Y Heo CY. Relationship between obesity and surgical complications after reduction mammaplasty: a systematic literature review and meta-analysis. Aesthet Surg J. 2017;37:308–15. 28207040 10.1093/asj/sjw189

[34] Madsen HJ Gillette RA Colborn KL. The association between obesity and postoperative outcomes in a broad surgical population: a 7-year American college of surgeons national surgical quality improvement analysis. Surgery. 2023;173:1213–9. 36872175 10.1016/j.surg.2023.02.001

[35] Valent AM DeArmond C Houston JM. Effect of post-cesarean delivery oral cephalexin and metronidazole on surgical site infection among obese women: a randomized clinical trial. JAMA. 2017;318:1026–34. 28975304 10.1001/jama.2017.10567 PMC5818802

[36] Cheadle WG. Risk factors for surgical site infection. Surg Infect (Larchmt). 2006;7(Suppl 1):S7–11. 16834549 10.1089/sur.2006.7.s1-7

[37] Huttunen R Syrjänen J. Obesity and the risk and outcome of infection. Int J Obes (Lond). 2013;37:333–40. 22546772 10.1038/ijo.2012.62

[38] Waisbren E Rosen H Bader AM Lipsitz SR Rogers SO Eriksson E. Percent body fat and prediction of surgical site infection. J Am Coll Surg. 2010;210:381–9. 20347729 10.1016/j.jamcollsurg.2010.01.004

[39] Lan G Xie M Lan J. Association and mediation between educational attainment and respiratory diseases: a Mendelian randomization study. Respir Res. 2024;25:115. 38448970 10.1186/s12931-024-02722-4 PMC10918882

[40] Cohen AK Syme SL. Education: a missed opportunity for public health intervention. Am J Public Health. 2013;103:997–1001. 23597373 10.2105/AJPH.2012.300993 PMC3698749

[41] Belot A Fowler H Njagi EN. Association between age, deprivation and specific comorbid conditions and the receipt of major surgery in patients with non-small cell lung cancer in England: a population-based study. Thorax. 2019;74:51–9. 30100577 10.1136/thoraxjnl-2017-211395

[42] Brundisini F Giacomini M DeJean D. Chronic disease patients’ experiences with accessing health care in rural and remote areas: a systematic review and qualitative meta-synthesis. Ont Health Technol Assess Ser. 2013;13:1–33. PMC3817950 24228078

[43] Collins PF Stratton RJ Kurukulaaratchy RJ Elia M. Influence of deprivation on health care use, health care costs, and mortality in COPD. Int J Chron Obstruct Pulmon Dis. 2018;13:1289–96. 29719384 10.2147/COPD.S157594 PMC5914553

